# Features of Retinal Neurogenesis as a Key Factor of Age-Related Neurodegeneration: Myth or Reality?

**DOI:** 10.3390/ijms22147373

**Published:** 2021-07-09

**Authors:** Darya V. Telegina, Oyuna S. Kozhevnikova, Anna K. Antonenko, Nataliya G. Kolosova

**Affiliations:** 1 Institute of Cytology and Genetics, Siberian Branch of Russian Academy of Sciences, 630090 Novosibirsk, Russia; oidopova@bionet.nsc.ru (O.S.K.); antonenko@bionet.nsc.ru (A.K.A.); kolosova@bionet.nsc.ru (N.G.K.); 2 Department of Natural Sciences, Novosibirsk State University, 630090 Novosibirsk, Russia

**Keywords:** retina, neurodegeneration, AMD, aging, neurogenesis, development, transcription factor

## Abstract

Age-related macular degeneration (AMD) is a complex multifactorial neurodegenerative disease that constitutes the most common cause of irreversible blindness in the elderly in the developed countries. Incomplete knowledge about its pathogenesis prevents the search for effective methods of prevention and treatment of AMD, primarily of its “dry” type which is by far the most common (90% of all AMD cases). In the recent years, AMD has become “younger”: late stages of the disease are now detected in relatively young people. It is known that AMD pathogenesis—according to the age-related structural and functional changes in the retina—is linked with inflammation, hypoxia, oxidative stress, mitochondrial dysfunction, and an impairment of neurotrophic support, but the mechanisms that trigger the conversion of normal age-related changes to the pathological process as well as the reason for early AMD development remain unclear. In the adult mammalian retina, de novo neurogenesis is very limited. Therefore, the structural and functional features that arise during its maturation and formation can exert long-term effects on further ontogenesis of this tissue. The aim of this review was to discuss possible contributions of the changes/disturbances in retinal neurogenesis to the early development of AMD.

## 1. Introduction

Age-related macular degeneration (AMD) is a complex multifactorial disease leading to irreversible loss of photoreceptors, retinal pigment epithelium (RPE) cell death, and pathological alterations of the Bruch’s membrane and the choriocapillaris in the macula. AMD is the main cause of irreversible vision loss in people over 60 years of age in the developed countries [1]. Effective methods of prevention and treatment of AMD are not available due to incomplete knowledge about AMD pathogenesis, which is based on retinal structural and functional changes that are characteristic of physiological aging [1]. It is reported that aside from inflammation, hypoxia, and oxidative stress [1], a major role in the development of AMD is played by RPE and glial dysfunction [2,3], cellular senescence [4] and immunosenescence [5], impaired autophagy [6], mitochondrial dysfunction [7], and impaired neurotrophic support [8,9]. On the basis of the clinical data, it is customary to distinguish between the “dry” and “wet” types of this disease. The “dry” (nonexudative) type represents 90% of AMD cases and is characterized by the presence of drusen in the macula, defects in the pigment epithelium and the choriocapillaris, and progressive loss of neurons [4]. The “wet” (exudative) type represents 10% of AMD cases and features the ingrowth of newly formed vessels through Bruch’s membrane defects under the RPE or the neuroepithelium. Pathological permeability of the newly formed vessels leads to retinal edema, exudates, and hemorrhages into the vitreous body and the retina, thereby eventually causing loss of vision [10].

In recent years, research and development of new therapies designed to increase vascular remodeling (such as intravitreal injections of vascular endothelial growth factor (anti-VEGF therapy)) radically changed the treatment of the “wet” AMD type, thus allowing physicians to make significant progress in this field [11]. Nonetheless, there is still no effective treatment for the “dry” type of AMD: current modalities can only slow down the progression of existing atrophy [12]. Lately, cell replacement therapy [13] and regeneration stimulation in the adult retina using special medicines have been being considered as new promising modalities, although causing heated debates in the scientific community [14,15,16].

All retinal neurodegenerative diseases are based on the decrease in metabolic and recovery processes and impairments of retinal microcirculation and structural organization [17]. The retina has a common basic organization among all vertebrate species, thus making it possible to use animals to study the mechanisms underlying the maintenance of the normal physiological structure of the retina and the pathogenesis of many diseases to apply the gained knowledge to the development of new treatments for these diseases in humans [18]. Such research is carried out mainly on rodents. It should be noted that retinopathies in rodent models are considerably different from classic AMD in humans, and the study of AMD is complicated by limitations of animal models. Rodents do not have the macula, nor do they have an area of high cone density analogous to the fovea [18]. In addition, rodents do not develop deposits at the base of the RPE that have a composition similar to that of drusen in humans, thereby perhaps reflecting a distinct manner in which lipids are transported across the RPE in rodents [18]. The inner limitation membrane and the Bruch’s membrane of rodents differ from the corresponding human structures which undergo topographic and age-related alterations not seen in mice and rats [19,20]. Furthermore, different mouse strains have distinct topographical and cone type-specific susceptibility to retinal degeneration [21,22].

It should be emphasized that today, there is no experimental model that emulates the whole pathophysiology of AMD. An ideal model should capture anatomical features and pathophysiological mechanisms, mimic the progression pattern, and be amenable to the evaluation of translational endpoints and treatment approaches [23]. Genetic variation has been shown to exert a strong influence on the development and progression of AMD as well as on the response to treatment among patients with AMD. Therefore, studies on different animal models are necessary for attributing signs of the disease to certain genetic variants. Various murine and rat models are used to simulate early [24], atrophic (“dry”) [25,26,27], and “wet” AMD [28]. Nonetheless, most models of “dry” AMD involve rapid retinal degeneration and therefore fail to recapitulate the progressive nature of this disease. On the other hand, there are rodent models with senescence acceleration that feature slow chronic degeneration of the retina, for example, OXYS rats [29] and senescence-accelerated mouse prone 8 (SAMP8) mice [30].

In spite of the limitations, in the biomedical research today, rodents (especially mice) are by far the most widely used species, mainly due to cost effectiveness, short reproduction cycles, and established and continuously emerging opportunities for genetic manipulations [31].

In the retina of adult mammals—in contrast to birds and amphibians—de novo neurogenesis and regenerative capacity are very limited [32]. In this regard, the structural and functional features specific to the period of maturation and formation of the retina may have long-term effects in ontogenesis, including greater predisposition to aging-associated diseases, thereby enhancing sensitivity of the retina to such universal triggers as inflammation and oxidative stress.

Research on the processes of histogenesis and on the beginning and end of the main stages of retinal development is necessary to understand the mechanisms of pathological processes that—by developing in the immature retina—can lead to a substantial change in its structure and functions in an adult organism. For example, some data suggest that the increased susceptibility to chronic disorders is more likely among the people born prematurely [33]. Most of them have retinopathy of prematurity: this pathology derives from the immaturity of retinal neurovascular structures and has a considerable long-term impact on vision [34]. Total retinal thickness in the fovea is greater in the people who were preterm low birth weight infants in comparison with full-term infants regardless of postnatal retinopathy of prematurity [35,36,37,38]. These changes last at least until young adulthood and may result in a reduced functional response of the retina [39], suggesting that retinal morphological and functional alterations may persist throughout the lifespan.

Only a few studies have addressed the relationship between birth weight and AMD. Newborns with low (<2500 g [40]) and high (>4000 g [41,42]) birth weight are at a higher AMD risk in adulthood as compared to normal birth weight newborns. It has been proposed that low or high body weight reflects an impairment of fetal development and a higher risk of eye diseases in adulthood.

The research on neurodegenerative diseases has traditionally been focused on later life stages. There is now growing evidence that they may be programmed during early development. The theory of “fetal developmental programming” [43,44] postulates that parental adversity and fetal factors permanently modify human systems of organs, including the nervous system, and in adulthood, predispose them to premature aging and to the related diseases of the brain [45]. This review was aimed at examining possible contributions of abnormalities of retinal neurogenesis to the pathogenesis of AMD.

## 2. Retina Development: Similarities and Differences between Humans and Laboratory Animals

**Figure 1:**
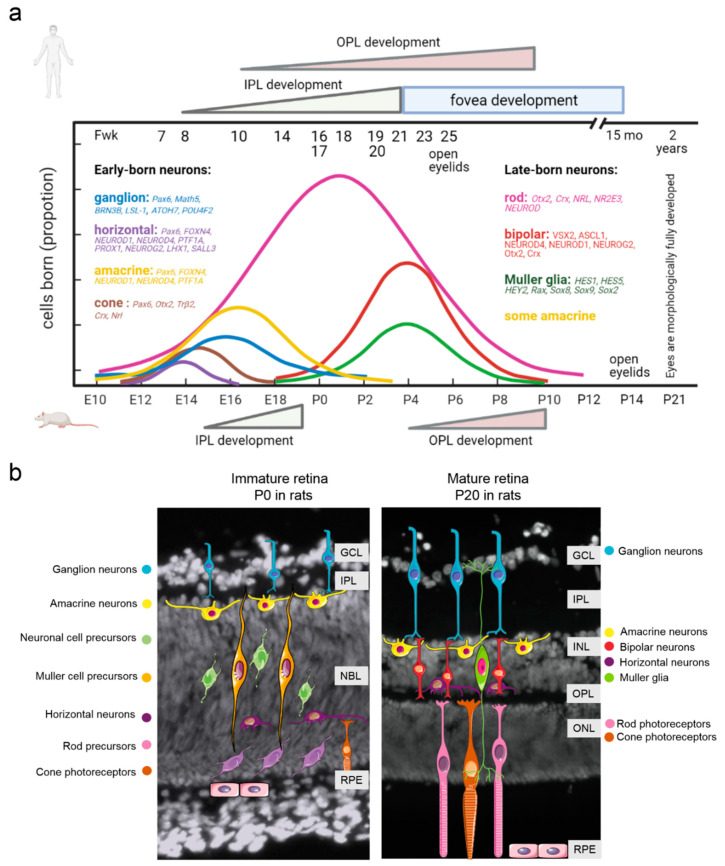
Time course of retinal development. (**a**) Comparative analysis between humans and rodents (mice and rats). The figure shows the transcription factors described in the body of the article. Adapted from [57,62] with permission. Neurogenesis in the human retina occurs during fetal development. The foveal pit appears at fetal week 25 and looks mature by 15 months [62]. The IPL emerges in the presumptive fovea at fetal week 8, reaches the eccentricity of the optic nerve by fetal week 12, and is present in both nasal and temporal peripheral edges by fetal week 18–21. The OPL first appears in the fetal week 11 fovea and reaches retinal edges by fetal week 30 [55]. In humans, eyelids open by fetal week 25 and morphologically full development of the retina is completed only at 2 years of age [58]. (**b**) At birth, the rat retina is still poorly developed and is composed of the GCL, a thin IPL, and a prominent densely packed neuroblast layer [52]. OPL formation occurs postnatally, between P4 and P10 [54], but IPL formation takes place during embryogenesis [65]. Rodents open their eyelids at the age of P14 and reach morphologically full retinal development at P20–P21 [58]. It is noteworthy that morphological formation of plexiform layers is not indicative of their functional maturation. Synaptogenesis continues long after birth in both humans and rodents [66]. NBL, neuroblast cell layer; GCL, ganglion cell layer; INL, inner nuclear layer; IPL, inner plexiform layer; OPL, outer plexiform layer; ONL, outer nuclear layer; RPE, retinal pigment epithelium; Fwk, fetal week.

Neurogenesis in the central and peripheral nervous systems includes three main stages: (1) formation of neurons from neural progenitor cells; (2) migration of neurons from proliferative zones to a site of permanent localization; and (3) differentiation and acquisition of molecular and morphological features [46]. All these steps are regulated in complicated ways by transcription factors, signaling pathways, and neurotrophic support [47]. The retina can be regarded as part of the brain and is characterized by highly conserved architecture across all relevant species [17]. The retina consists of six types of neurons (with ~50 subtypes) and one type of glia, all derived from multipotent progenitor retinal cells [48]. During retinogenesis, these neurons are formed by three nuclear layers, which are separated by two plexiform (synaptic) layers. The ganglion cell layer (GCL) contains nuclei of ganglion cells, whose axons become optic nerve fibers, and some displaced amacrine cells; the inner nuclear layer (INL) contains nuclei of amacrine cells, bipolar cells, horizontal cells, and Müller glia; whereas the outer nuclear layer (ONL) contains cell bodies of photoreceptors. Synapses between bipolar cell axons and dendrites of ganglion and amacrine cells constitute the inner plexiform layer (IPL). The outer plexiform layer (OPL) is composed of synapses representing presynaptic horizontal and photoreceptor cells and postsynaptic bipolar cells [17] (Figure 1).

The retina is a convenient model for the investigation of neurogenesis because retinal cell genesis is highly conserved and the development of all types of neurons and glia presents a well-defined time course [49]. Nevertheless, many questions remain unanswered. In particular, there is no consensus on how the formation of different cell types proceeds exactly. Most scientists believe that retinal progenitor cells in vertebrates can give rise to different retinal cell types in a stochastic manner with a probabilistic bias for some cell types that change during development [48].

In the retina of rats and mice, neurogenesis proceeds in two waves: in the early phase, beginning on embryonic day 10 (E10), ganglion neurons, horizontal cells, and cone photoreceptor neurons come into being. In the late phase during early postnatal development (postnatal days 0–12; P0–P12), bipolar neurons, Müller cells, and rod photoreceptor neurons are formed. Amacrine cells with various neurotransmitters are formed during both early and late phases [50]. The development of the retina proceeds from the central retina toward the peripheral retina and from the GCL to the ONL. Therefore, ganglion neurons in the central retina are the first retinal cells to start differentiating [51].

Rodents are born with closed eyes that contain the retina with well-formed GCL, IPL, and neuroblast layer (which includes retinal progenitor cells) [52] (Figure 1b). Amacrine cells and bipolar cells project presynaptic dendrites to the IPL, in which they synapse with retinal ganglion cells. OPL formation lags well behind the IPL formation in the eye owing to later formation of photoreceptors and bipolar cells [53]. In rodents, OPL formation occurs postnatally before the eyes open [54]. In humans, the OPL develops slowly and reaches retinal edges by fetal week 30 [55]. The formation of synaptic connections between neural dendrites and axons is the step crucial for the proper functioning of retinal neural circuits. Fan et al. showed that the time before P14, especially between P0 and P14, represents a critical period of retinal development. Murine eye opening takes place in that period, suggesting that cell differentiation and synaptic formation result in the visual function [52]. It must be noted that the opening of the eyes in rodents does not mean full functional maturity of the retina. In rabbits and rats, amplitudes of retinal light responses measured by electroretinography continuously increase in the first month after birth and reach the adult level between P30 and P40 [56] (Figure 1).

Recently, similar retinal time courses were described for the developing human retina on the basis of high-throughput RNA sequencing data [57]. Nevertheless, the development of the human retina, just as that of other primates, has a number of substantial differences from the corresponding rodent process. The bulk of highly complex retinal development occurs between gestational week 24 and the age of 3 to 4 months, when the optic nerve becomes fully myelinated [58]. Retinogenesis in humans and structural formation of all the retinal layers take place during fetal development [59]. The peripheral retina of humans shares laminated cellular organization and basic developmental events with those of other vertebrates [57]. By contrast, the human retina contains a central “rod-free” zone termed the macula, which is required for high visual acuity and color vision. The macula is subdivided into the umbo, the foveola, the foveal avascular zone, the fovea, the parafovea, and the perifovea [60]. The fovea is a pitted invagination in the inner retina that contains especially large numbers of photoreceptors and neurons and provides the highest visual resolution [61]. The macula develops and matures much earlier than the rest of the retina: macula formation begins between the 11th week and the 13th week of gestational age [59,62]. The fovea, i.e., the inner part of the macula, develops starting from fetal week 20–22, and this development continues into early childhood [62]. Newborns have underdeveloped outer segments in the fovea [62]. At birth, rod cells are better developed than cone cells. As a consequence, neonates primarily see shades of gray. Color discrimination starts at 3 months of age when cones mature [63]. With age, cone outer segments become longer, but full maturation of the human retina does not occur until early adolescence [59]. Impaired maturation of photoreceptors in childhood can cause a number of diseases of the retina in children.

Using high-throughput RNA sequencing data, Hoshino et al. detected temporally correlating gene expression patterns between the human and mouse retinas. At early stages of development, tighter temporal correlation was observed between mouse (E12–E14) and human genes (D52–D57, fetal week 7–8). The E16–P0 stage in the developing mouse retina was found to be concordant with the human fetal retina from D67 to D107. In this period, the human peripheral retina exhibited both cell proliferation and differentiation (as did the mouse retina), but foveal neurogenesis and lamination were complete. Expression of genes during D115–D136 in the human fetal retina correlated with that in the mouse P2–P4 retina. None of the human samples clustered with the maturing P6–P28 mouse retina probably because the fetal peripheral retina had not yet attained the developmental maturity of the late postnatal murine retina [57] (Figure 1). The genes with the highest degree of correlation between these species were related to the G2–M cell cycle transition (the correlated genes were downregulated) and photoreceptor development (the correlated genes were upregulated). The set of “anticorrelated” genes, i.e., of those featuring opposite patterns in the developing human and mouse retinas, did not exhibit enrichment with any specific Gene Ontology category [57].

Another study revealed that epigenetic changes during retinal development are strongly conserved between mice and humans, with DNA methylation changes being the least conserved (7–8%), chromHMM being more highly conserved (45–56%), and developmental stage-specific superenhancers being the most conserved (62%) [64].

The above studies have shown strong conservation (across species) of expression patterns of the genes involved in retinal development, thereby allowing us to gain in-depth knowledge about these mechanisms.

There is another important factor affecting the research into neurogenesis and correlations between humans and experimental animals. Neurogenesis is also influenced by such disorders as albinism. Albinism, i.e., hypopigmentation resulting from a reduction in melanin synthesis, is a rare genetic disease in humans but is often seen in various experimental models based on mice and rats. Abnormal retinal development is also evident in this disease and causes foveal hypoplasia [67], a reduction in the number of ipsilateral retinal ganglion cells [68] and photoreceptors [69], and an abnormal distribution of rods [70]. These problems lead to visual impairments such as poor visual acuity, photophobia, nystagmus, and abnormal binocular vision [71]. All these features of neurogenesis should be taken into account by investigators to interpret experimental results and extrapolate the data obtained in laboratory animals to humans.

## 3. The Transcriptional Network Regulating Retinal Neurogenesis

All stages of neurogenesis are subject to complex regulation by extrinsic (NOTCH signaling, BMP signaling, and provision of neurotrophic support) and intrinsic factors (transcription factors) [72]. Transcription factors, in accordance with a genetic program and/or in response to an external stimulus, initiate or suppress the transcription of certain genes, thereby driving changes in cell morphology, cell differentiation, morphogenesis, organogenesis, and other parameters. As a consequence, the interaction of various combinations of transcription factors causes the formation of various types of neurons from pluripotent progenitors. Experimental data indicate the presence of a pool of oligopotent progenitors of neurons in a differentiation-associated embryonic period (early-born progenitors: an early type of cells) [73]. The early-born neurons express the *Pax6* gene, whose product is a highly conserved vertebrate transcription factor required for retina formation. Mutation of *Pax6* or its overexpression leads to several pathological phenotypes: cataract, aniridia, or the absence of an eye in mice and humans [74]. When Pax6 is inactivated in all retinal cells starting from E15.5, the numbers of retinal ganglion cells, amacrine cells, horizontal cells, and photoreceptors drastically decrease as compared to control mice [75]. Moreover, the expression of many genes that are essential for retinal development is severely affected by PAX6 loss. In this regard, the most important genes are *NeuroD1*, *NeuroD4*, *Nr2e3*, *Rp1*, *Prph2*, and *Rho*, whose products are involved in fate specification of retinal neurons [75]. On the other hand, *Pax6* overexpression can yield aberrant axonal patterns where axons fail to navigate toward the optic chiasm [76]. A recent report showed that *Pax6* expression after fate specification of retinal ganglion cells is necessary for the regulation of expression of axon guidance genes and most importantly for the maintenance of conducive extracellular matrix molecules through which the nascent axons get guided and fasciculate to reach the optic disc [75].

**Figure 2:**
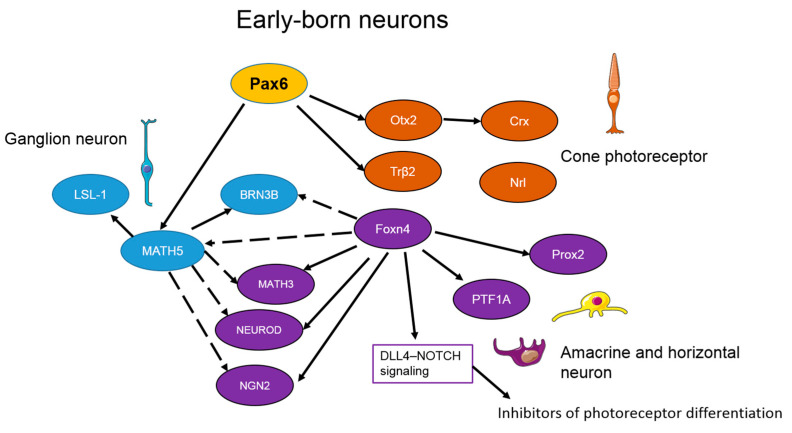
The proposed gene regulatory network of the early-born neurons’ genesis. The figure shows the transcription factors described in the body of the article. Solid arrows indicate activation, dotted arrows—inhibition.

Persistent expression of *Pax6* induces *Math5*, which takes part in the regulation of differentiation of ganglion neurons [75]. It is suggested that the regulation by MATH5 is implemented in two pathways. At first, MATH5 activates a downregulated signaling network that controls the differentiation and development of ganglion cells [77,78]. Next, MATH5 downregulates the genes encoding other proneuronal transcription factors, such as MATH3, NEUROD, and NGN2, which are involved in the differentiation of other types of retinal neurons [49,79]. Deletion of *Math5* causes the loss of 80% of retinal ganglion neurons and increases the formation of amacrine and horizontal cells, cones, and Müller cells [47,79]. MATH5-upregulated transcription factors BRN3B (POU4F2) and LSL-1 are required for further differentiation of ganglion cells, and MATH5 expression decreases afterwards [80]. PAX6 can also induce *Otx2* and *Trβ2* in some oligopotent progenitors, yielding cone-like differentiation of photoreceptors (Figure 2) [81]. In rodents, cones express both short wavelength (S)-sensitive opsin and medium wavelength (M)-sensitive opsin, while rods express only rhodopsin [60]. OTX2 mediates the differentiation of progenitor cells into photoreceptors by launching the expression of the CRX transcription factor (cone rod homeobox). CRX promotes the expression of M- and S-opsins and, in a synergistic interaction with NRL, activates the rhodopsin enzyme [49,82]. In humans, mutations in *Crx* cause several diseases characterized by the loss of photoreceptors: cone–rod dystrophy, retinitis pigmentosa, and Leber amaurosis [47,83].

Horizontal and amacrine cells are present in the pool of bipotent progenitors and are early-born neurons, too [73]. Such transcription factors as FOXN4, NEUROD1, NEUROD4, and PTF1A participate in their specification (Figure 2). Inactivation of *Foxn4* leads to the loss of all horizontal and most amacrine cells; instead of them, ganglion cells and photoreceptors are generated [47]. Similar phenotypic characteristics are seen in *Ptf1a* knockout mice [84]. The transcription factor FOXN4 enhances the expression of NEUROD1, NEUROD4, and PTF1A, which are necessary for the differentiation of amacrine and ganglion neurons [72]. FOXN4 directly inhibits ATOH7 (MATH5) and POU4F2 (BRN3B) (required for ganglion neurons) and activates the DLL4–NOTCH signaling, which launches inhibitors of photoreceptor differentiation [85]. FOXN4 is essential for the genesis of horizontal cells [86]. For instance, FOXN4 activates many transcription factors, such as PTF1A, PROX1, NEUROD1, NEUROD4, and NEUROG2, which interact with each other to bring about the differentiation of this cell type (Figure 2) [47]. Knockouts of genes *NeuroD1*, *NeuroD4*, and *Neurog2* have been shown to completely eliminate horizontal cells, just as in *Ptf1a* knockout mice [87]. In addition, transcription factors LHX1 and SALL3 control the migration and position of horizontal cells. LHX1 and SALL3 inactivation leads to a mutant phenotype and displacement of horizontal cells in the retina [88,89].

**Figure 3:**
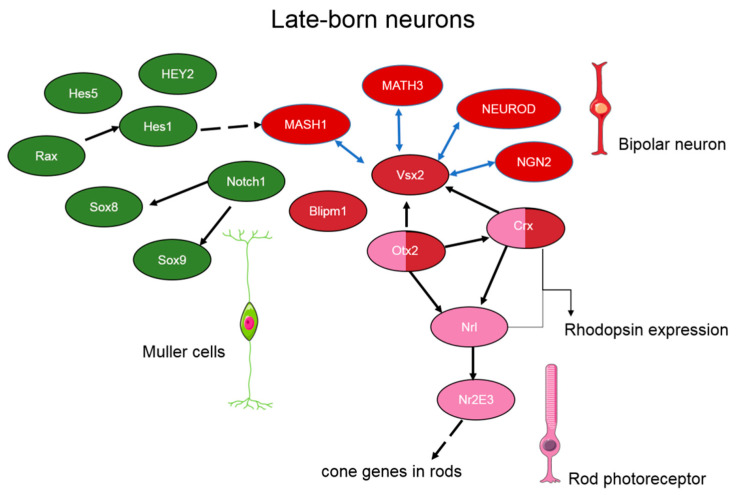
The proposed gene regulatory network of the late-born neurons’ genesis. The figure shows the transcription factors described in the body of the article. Solid arrows indicate activation, dotted arrows—inhibition, blue arrows—a synergistic interaction.

Late-born progenitor (LBP) cell types (Müller cells, bipolar neurons, and rods) differentiate in the first weeks of postnatal development in rodents and are thought to have a pool of common oligopotent progenitors [73]. Nonetheless, the general transcription factors that control LBP differentiation are still not known. The direction of differentiation in postnatal retinal neurogenesis depends on the expression of *Vsx2*, *Otx2*, *Blimp1*, and *Notch1* (Figure 3). For instance, it is reported that the synergistic interaction of transcription factor VSX2 and bHLH transcription factors ASCL1/MASH1, NEUROD4/MATH3, NEUROD1, and NEUROG2/NGN2 results in the development of bipolar neurons [47]. *Vsx2* mutations cause a complete loss of bipolar cells, thus increasing the population of photoreceptor cells and Müller cells [90]. The differentiation of bipolar cells requires OTX2 and CRX, too. A significant decrease in the population of bipolar cells has been demonstrated in *Otx2*/*Crx* double-knockout mice [91]. OTX2 and CRX may control bipolar cell differentiation through direct binding to *cis*-regulatory sequences of *Vsx2* [90] (Figure 3). In addition, two proteins are known to suppress CRX functions and promote retinal degeneration: ataxin 7 and BAF [91].

Müller cells are the only type of glia that derive from retinal progenitor cells [73]. Therefore, the factors determining the selection of progenitor cell fate between gliogenesis and neurogenesis are critical for retinal development. Activation of the NOTCH signaling pathway is primarily required for the differentiation of Müller cells [92]. During early Müller cell differentiation, transcription factors HES1, HES5, and HEY2 are expressed, but later these factors are restricted to Müller cells, and their overexpression strongly promotes Müller cell fate at the expense of neurons [93]. Conversely, *Hes5* inactivation decreases the formation of Müller cells, which is promoted by both overexpression of NOTCH1 and increased expression of its effectors: HESR2 or HES1 [93]. Similarly to the HES transcription factors, RAX determines Müller cell fate and does so partly by directly inducing *Hes1* [70]. HES1, in turn, represses the proneuronal *Mash1* gene (Ascl1) [94]. NOTCH1 also enhances the expression of *Sox8* and *Sox9* [95]. Inactivation of *Sox9* in a developing retina leads to the loss of Müller cells, and a *Sox8* knockout decreases the glial population (Figure 3) [85,95]. SOX2 is needed for Müller cell specification because SOX2 misexpression in postnatal retinal progenitor cells promotes Müller and amacrine cell fates at the expense of rod cells [96].

Cone differentiation is mediated by CRX, NRL, and NR2E3. For example, CRX is first expressed at early stages of retinal development and retains its expression in mature photoreceptors [50]. CRX activates a promoter of rhodopsin and stimulates rhodopsin expression in cones that acts synergistically with NRL. CRX is also able to activate opsin in cones [87]. CRX inhibitors ataxin 7 and BAF contribute to photoreceptor degeneration [97]. The transcription factor NRL is mainly expressed in rods and promotes the expression of rhodopsin [98]. OTX2 and CRX directly bind to an enhancer of *Nrl* and activate its expression [99]. In turn, NRL induces numerous rod-specific genes and downregulates cone-specific genes, partly through direct regulation of the expression of a nuclear receptor gene, *Nr2e3* [47]. In humans, mutations in the *NRL* gene are associated with autosomal dominant retinitis pigmentosa (Figure 3) [100,101]. NR2E3 acts as a repressor of cone genes in rods and directly interacts with CRX, thereby enhancing rhodopsin expression [102]. Another major well-studied transcription factor regulating the differentiation of photoreceptors is NEUROD. This factor is first expressed in embryogenesis and retains its expression in the mature retina. In the absence of NEUROD, the number of rods diminishes, while the number of bipolar cells increases proportionally [87]. Incorrect expression of *NeuroD* not only blocks gliogenesis, but also promotes rod differentiation and suppresses differentiation into bipolar cells. NEUROD is also required for constant expression of TRβ2, a protein important for cone development (Figure 3) [103].

Recent research indicates that retinal neurogenesis is a labile process. Disturbances in the differentiation of some cell types resulting in a proportional increase in the population of other neurons can cause a change in functions and adaptive capabilities [73].

To summarize, it should be noted that in the investigation of molecular mechanisms underlying retinal neurogenesis, there has been substantial progress in the last decade. Nevertheless, many key questions remain unanswered, in particular, the question of the possible contribution of changes/disturbances in retinal neurogenesis to the onset of AMD and aging.

## 4. The Role of Transcription Factors during Retinal Aging and Age-Related Degeneration

Research on expression patterns and regulatory functions of transcription factors has been focused on developmental stages alone, and little is known about the roles of transcription factors in the adult, aged, or degenerative mammalian retina.

Homeobox genes (e.g., *Otx2*, *Crx*, *Pax6*, and *Sox2*) play the key role in many parameters of retinal development, including eye and retina formation, and in subsequent differentiation of neural tissue [104]. Due to the large contribution of these transcription factors and their cascade activation during vertebrate retina development, mutations in homeobox genes give rise to many ophthalmic abnormalities in humans. In humans, mutations in homeobox genes are rare and constitute primary etiology of some retinal diseases that are characterized by inherited progressive degeneration of photoreceptors, e.g., cone–rod degeneration, retinitis pigmentosa, and Leber congenital amaurosis [47].

In the mature retina, *Otx2* is expressed in the RPE, photoreceptors, and bipolar cells, whereas its constant expression is necessary for homeostasis in the retina [105]. Studies in mice have revealed that the severity of ophthalmic disorders is inversely proportional to OTX2 activity [106]. For instance, in mice with a single mutant *Otx2AA*/*GFP* allele (20% activity in comparison with the wild type), the retina is significantly thinner. In mice with 50% or 70% OTX2 activity (compared with the control), an INL consisting of bipolar, amacrine, and horizontal cells thins with age. Additionally, by the age of 60 days, these mice exhibit functional defects, a decrease in visual acuity, abnormal bipolar cell activity, and progressive cell death, the severity of which corresponds to the level of the assumed OTX2 activity [106]. Moreover, there is evidence that a conditional *Otx2* knockout yields adult mice with RPE dysfunction followed by progressive and complete loss of photoreceptors; these alterations resemble degeneration in patients with AMD [107]. Studies involving intravitreal injection of the OTX2 protein in mice have shown that the OTX2 protein amount increases in almost all retinal layers, especially in ganglion and bipolar cells and photoreceptors. These injections promote the survival of neurons and may have therapeutic value in some severe pathologies, such as congenital night blindness, glaucoma, and AMD [108]. In the adult retina, CRX regulates the expression of many photoreceptor-specific genes including opsins. Mutations in *Crx* cause various retina degeneration phenotypes in humans [35]. In *Crx*^−/^^−^ knockout mice, the development of photoreceptors and synaptic contacts is strongly impaired, and there is decreased OPL thickness and changed expression of genes associated with calcium homeostasis [109]. It is worth noting that the interaction of OTX2 and CRX is a key event not only for retinal neurogenesis, but also for synaptogenesis. For example, transcription factor CRX, along with another transcription factor (NRL), modulates the formation of presynaptic endings of photoreceptors in the ONL [110]. OTX2 also controls synaptogenesis by upregulating the pikachurin protein, which binds to β-dystroglycan in the presynaptic region of photoreceptors [111]. Therefore, mutations and changes in the activity of OTX2 and CRX during the formation of retinal neurons can have a significant impact on further ontogenesis of this tissue and a long-term effect on eyesight.

Other development-related transcription factors, SOX2 and PAX6, are of interest for the research on neurodegenerative diseases. *Pax6* and *Sox2* heterozygous mutations in humans cause a characteristic spectrum of nervous system abnormalities that include eye defects such as microphthalmia (small eyes) or anophthalmia (absent eye) [112]. SOX2 plays an essential role in progenitor cell maintenance in the developing and adult central nervous system [113].

Ablation of *Sox2* at the height of postnatal genesis of Müller cells in mice (P5) results in disorganization of processes of Müller glia in the IPL and mislocalized cell bodies in the nuclear layers; this feature becomes most conspicuous at P25. In *Sox2* mutants, adherens junctions are shorter and not oriented correctly [114]. This disorganization is concurrent with neural retina thinning and an impairment of neuronal processes in the IPL and the OPL. Moreover, there is a decrease in the b-wave amplitude in both young *Sox2* mutant mice and aged *Sox2GFP* mice; therefore, the retina function is affected negatively [114,115]. SOX2 plays an essential role in the maintenance of the structural organization of the postnatal retina and in the quiescence of nascent Müller cells. Loss of SOX2 forces Müller cells to aberrantly divide into a pair of postmitotic daughter cells, thereby causing Müller glial cell depletion and retinal degeneration [116]. Moreover, a decline in *Sox2* expression with age is seen in Müller cells and in amacrine and ganglion neurons, and this decrease impairs the activity of these cells [115]. In that study, investigators found that aged *Sox2GFP* mice feature an impaired visual function, indicating the critical role that Sox2 plays in age-related vision maintenance [115]. Astrocytic loss of *Sox2* affects vascular architecture during maturity [117]. Altogether, these findings indicate that Sox2 is required for the maintenance of visual information transmission and suggest that the decline in *Sox2* expression is responsible for retinal cell aging and age-related vision loss. There is a hypothesis that SOX2 levels are a predictor of disease in the retina [118]. This theory supports the idea that once the level of SOX2 decreases down to phenotypically manifested levels (a threshold defined as 40% of the norm), disorders may appear [118].

PAX6, the master regulator of eye development, remains distinctly expressed in the aging mammalian retina; these data suggest a distinct role of PAX6 in the retina after the completion of eye morphogenesis. PAX6 has been found in both healthy and degenerative adult mammalian retinas. PAX6 expression in the retina is detectable up to 79 years of age in donors and is predominantly localized in the GCL and the inner part of the INL [119]. In mouse retinas, PAX6 protein levels are high at P5, decrease to intermediate levels at P21, and remain constant thereafter at least until P428 [120]. In two models of retinal degeneration (rd10 mice and light-induced retinal degeneration), it has been revealed that photoreceptor injury causes PAX6-positive Müller cell nuclei to relocate from their normal position in the middle of the INL toward the outer INL and even into the inner part of the ONL. The expression of several molecular markers suggests that these Müller cells attempt to reenter the cell cycle but fail to do so, resulting in non-proliferative gliosis [121]. In that study, although the Müller cell nucleus translocation occurred in both models of photoreceptor degeneration, total PAX6 protein expression was upregulated only in the retina of rd10 mice, not after light-induced degeneration [121]. This result may be related to the speed of neuron degeneration. Rd10 mice are characterized by slow photoreceptor degeneration, whereas light exposure induces acute degeneration along with toxic stress [121].

Another research group has shown that in rd1 mice (a common model of inherited retinal degeneration), the decrease in the PAX6 protein expression is accompanied by a peak of photoreceptor apoptosis (between P10 and P14). Immunohistochemical analysis revealed that PAX6 is localized in the ganglion and in the bipolar cell layer of the retina, but there are no PAX6-expressing cells in outer layers [119]. Those authors speculated that PAX6 has a protective effect against photoreceptor death. In the retina of wild-type (C3H background) mice, exposure to bright light decreases PAX6 expression. Conversely, PAX6 expression is higher in *c-Fos* knockout mice, which manifest resistance to light-induced retinal degeneration [119]. These findings may be related to a remodeling process involving Müller cells. It has been suggested that PAX6 exerts protective action in a degenerating outer retina through activation of Müller glial cells, which may be capable of producing retinal progenitor cells [119]. Pirmardan et al. investigated whether PAX6 overexpression can promote cell proliferation in the retina. They did not detect any changes in Ki67 expression after PAX6 overexpression but detected SOX2 expression in ONL cells after neurotoxic injury [122]. They registered SOX2 expression in all retina layers including photoreceptors and the GCL after 30 days of NMDA administration. Expression of stemness marker SOX2 indicates a high plasticity potential of retinal cells and highlights the involvement of the microenvironment and extracellular signaling in the acquisition of new phenotypes [122].

Lately, one of the challenging fields in biology is the use of an intrinsic regeneration potential in damaged organs. Investigation into the roles of transcription factors during aging and age-related diseases is a promising area for designing new treatments for degenerative retinal diseases.

## 5. Stimulation of Neurogenesis as a New Strategy for the Treatment of Degenerative Diseases of the Mammalian Retina

As described above, adult retinogenesis in mammals is very limited. Nevertheless, the retina has a possible endogenous cell resource for neurogenesis and regeneration: the ciliary body, the iris, Müller cells, and the RPE (reviewed in [123]). All these cells have substantial advantages over foreign cells transplanted for retinal repair because of their autologous origin. On the other hand, the most promising cells for retinal regeneration are Müller cells, which are discussed in detail below.

Müller cells are later-born differentiated radial-like glial cells that span the entire thickness of the retina. Their somata are localized in the INL, and their processes penetrate all layers of the retina, thereby promoting contacts between neighboring neurons during development and participating in the formation of external and internal limiting membranes [3]. Under normal homeostatic conditions in the adult retina, Müller glial cells ensure healthy retinal function by maintaining retinal architecture and by providing trophic support to neurons. In contrast, during aging and in pathological conditions (e.g., inflammation, oxidative stress, or injury), Müller glia undergo a series of changes and reach an activated state known as gliosis. The specific feature of reactive gliosis is overexpression of intermediate filaments, such as vimentin, nestin, and GFAP, and a hypertrophic morphology of reactive Müller cells [3]. Activated Müller cells also sometimes manifest reentry into the cell cycle and proliferation, giving rise to a glial scar, which next aggravates the damage at the late stage of retinal injury [3]. Furthermore, Müller cells take part in cellular remodeling in the vertebrate retina after its damage or detachment [124]. Retinal remodeling is a phenomenon consequent to photoreceptor degeneration consisting of a series of alterations in retinal metabolism, receptor expression, and neuronal network topologies, with eventual cell death and formation of aberrant synapses. During retinal degeneration, Müller cells are among the first to respond to stress [124]. Retinal remodeling is one of the reasons for the failures of cell-based therapies of retinal degeneration. Activated Müller cells in mice transiently express cell cycle regulators and neurogenic factors, and these cells rapidly return to quiescence [125]. This phenomenon is mediated by a dedicated gene regulatory network that includes NFI factors, which are upregulated at later stages after injury and return reactive Müller glia to a resting state in mice.

There is evidence that a small percentage of Müller glia reenter the mitotic cycle after acute damage by NMDA, MNU, NaIO_3_, ouabain, or α-aminoadipate; a subtoxic dose of glutamate; or excessive light in rats and mice [126]. Mitogens such as EGF or FGF2 and insulin significantly increase Müller glia proliferation after damage [127].

The reprogramming of Müller cells into functional neurons in adult mammals is a promising treatment of retinal neurodegenerative diseases. On the other hand, multiple independent mechanisms strongly repress neurogenic competence in mammals, making it a challenge to experimentally identify the regulators of Müller glia reprogramming. Nevertheless, there are some studies on Müller cell reprogramming in vivo in mice and rats.

When expressed in neural progenitor cells, proneural transcription factor ASCL1 promotes cell cycle exit and neuronal differentiation. In mammals, *Ascl1* is expressed in retinal progenitors and is needed for the development of rods and bipolar cells [128]. In the mature retina of mice, *Ascl1* is not expressed in Müller glia, whereas after damage or in disease models, mouse Müller cells do not show spontaneous upregulation of *Ascl1* [129]. Nevertheless, ectopic expression of *Ascl1* in adult Müller glia in the undamaged retina does not overtly affect their phenotype. When the retina is damaged in young mice, ASCL1-expressing glia initiate a response that resembles early stages of retinal regeneration in zebrafish. By P16, mouse Müller cells lose neurogenic capacity despite *Ascl1* overexpression [129]. In adult mice, *Ascl1* overexpression in Müller glia is no longer sufficient for inducing the neurogenic potential, even in the presence of damage [130]. Further research has revealed that overexpression of ASCL1, together with a histone deacetylase inhibitor, enables the formation of neurons from Müller cells in adult mice after retinal injury (NMDA damage) [130]. This combination predominantly gives rise to bipolar neurons derived from Müller cells but not from rod (or ganglion) cells [130]. This finding may be explained by a loss of neurogenic genes and of their accessible motifs during Müller glia maturation. Within bipolar cell-specific accessible regions in Müller cells, bHLH motifs are more highly enriched than within rod-specific accessible regions. Furthermore, overexpression of ASCL1 results in its binding to a greater number of bipolar cell-specific accessible regions than of rod-specific regions [131]. These data suggest that a reprogramming strategy involving ASCL1 alone is unlikely to regenerate rod photoreceptors and that additional transcription factors may be needed.

Downregulation of RNA-binding protein PTBP1 in the adult murine retina via in vivo viral delivery of a recently developed RNA-targeting CRISPR system, CasRx, promotes conversion of Müller glia into retinal ganglion cells with high effectiveness [132]. By contrast, no significant difference in *Ptbp1* expression is observed between control and neurogenic Nfia/b/x-deficient murine Müller glia. These observations do not support the hypothesis that the dynamic expression of PTBP1 directly regulates Müller glia reprogramming [125].

Webster et al. demonstrated that an agonist (PNU-282987) of α7 nicotinic acetylcholine receptor can induce Müller cells to form neurons after damage. In rats, topical application of PNU-282987-containing eye drops drives cell cycle reentry of vimentin-positive Müller glia and the production of nestin-positive retinal progenitor cells as evidenced by labeling with bromodeoxyuridine (BrdU) and proliferating cell nuclear antigen (PCNA). Moreover, the effects of PNU-282987 were dose-dependent and, when followed over time, yielded persistent BrdU labeling in cells within all the retinal layers and dramatic accumulation of BrdU-labeled retinal ganglion cells [14]. More detailed research suggests that PNU-282987 does not directly affect Müller glia but rather acts indirectly by triggering α7 nicotinic acetylcholine receptors in the RPE. Those authors suspect that after PNU-282987 treatment, a signaling molecule (or molecules) is released from the RPE and launches cell cycle reentry in Müller glia along with subsequent formation of retinal neurons in adult mammals [16].

## 6. Conclusions

Growing evidence suggests that longevity and the risk of age-related diseases may be programmed early in life. Prenatal and early postnatal periods are a crucial develop-mental window during which adverse factors can exert a lasting influence on the epigenome and gene expression throughout the lifespan [43,44,45]. In this review, we discussed possible contributions of changes/disturbances of retinal neurogenesis to early development of AMD. Even though little research has been directly focused on the possibility that preterm survivors experience accelerated biological aging, recent studies indicate that the risk of AMD can be affected by the factors acting at an early age, during the final stages of postnatal retinal maturation. Such risk factors include high, low, or extremely low birth weight [40,41,42] and the formation of aberrant neural circuits under the influence of genetic and/or environmental factors. Given the increased rates and early emergence of chronic illnesses observed in the individuals born with extremely low birth weight, the latter may confer a premature aging phenotype characterized by accelerated cellular senescence and the development of diseases typically associated with old age [133]. The mechanisms and potential substrates of these lasting effects are yet to be elucidated. We can speculate that early development of neurodegenerative diseases such as AMD most likely awaits those who have the abovementioned disorders. Understanding the impact of retinal development anomalies on the pathophysiology of AMD should help to devise novel therapeutics and prophylactic measures against AMD and other retinal disorders.
